# Proceedings of the Dental Society of the State of Maryland

**Published:** 1868-09

**Authors:** S. M. Field

**Affiliations:** Rec. Sec.


### ARTICLE VII.

### *Proceedings of the Dental Society of the State of Maryland.*

A meeting of this association was held at its rooms, May 28th,. Vice-president, Dr. Bean in the Chair.

After the transaction of the usual business on the meeting of the association, Dr. E. P. Keech called attention to an anomalous case which had recently come under his notice. It was that of a young girl, about twelve years old, in whose mouth the two temporary superior central incisors, were replaced by two teeth, which, in size and shape, closely resembled the third molars or wisdom teeth. Dr. K. exhibited the teeth and a plaster cast of the mouth, taken before they were extracted.

Dr. J. B. Bean read an interesting paper on, "A method of filling large shallow cavities on the labial surface of the incisor teeth."

The propositions of Dr. Arthur were then taken up, and after some discussion, the 1st proposition was slightly changed to read as follows:—

1st.—That caries of the teeth of the majority of children of the better classes of people in the United States at the present day, will certainly occur, sooner or later, on proximate surfaces of all the teeth, except the inferior incisors.

A vote was then taken by yeas and nays on each proposition, separately, and resulted in their unanimous endorsement by the members present, except the last proposition, which was laid over until the next meeting, in order to afford time for its further discussion.

At the June meeting of the association, the discussion of the 5th, proposition of Dr. Arthur was laid over by his request, until the October meeting.

S. M. Field, *Rec. Sec.*

